# FLIC: High-Throughput, Continuous Analysis of Feeding Behaviors in *Drosophila*

**DOI:** 10.1371/journal.pone.0101107

**Published:** 2014-06-30

**Authors:** Jennifer Ro, Zachary M. Harvanek, Scott D. Pletcher

**Affiliations:** 1 Cellular and Molecular Biology Program, University of Michigan, Ann Arbor, Michigan, United States of America; 2 Department of Molecular and Integrative Physiology and Geriatrics Center, University of Michigan, Ann Arbor, Michigan, United States of America; 3 Medical Scientist Training Program, University of Michigan, Ann Arbor, Michigan, United States of America; University of Houston, United States of America

## Abstract

We present a complete hardware and software system for collecting and quantifying continuous measures of feeding behaviors in the fruit fly, *Drosophila melanogaster*. The FLIC (Fly Liquid-Food Interaction Counter) detects analog electronic signals as brief as 50 µs that occur when a fly makes physical contact with liquid food. Signal characteristics effectively distinguish between different types of behaviors, such as feeding and tasting events. The FLIC system performs as well or better than popular methods for simple assays, and it provides an unprecedented opportunity to study novel components of feeding behavior, such as time-dependent changes in food preference and individual levels of motivation and hunger. Furthermore, FLIC experiments can persist indefinitely without disturbance, and we highlight this ability by establishing a detailed picture of circadian feeding behaviors in the fly. We believe that the FLIC system will work hand-in-hand with modern molecular techniques to facilitate mechanistic studies of feeding behaviors in *Drosophila* using modern, high-throughput technologies.

## Introduction

The ascent of the fruit fly, *Drosophila melanogaster*, as one of the most powerful model systems in which to dissect neural mechanisms of complex behavior has uncovered a need for innovation at the roots of the science. Technical advances in neurobiology have outpaced those that facilitate basic observation. Consequently, challenges identified as recently as five years ago as primary obstacles to discovery, such as the ability to temporally manipulate the expression of genes in specific brain regions or to alter the excitatory properties of individual neurons, have become standard practice [1]. In contrast, many experimental procedures that have been used for decades to characterize behaviors such as courtship, locomotor activity, and circadian rhythm have proven less than ideal for modern analysis. This is either because they fail to capture subtleties in the behavior that were not previously recognized or because they are not easily “scaled-up” and automated for genetic or pharmacological screens.

Measurement of fly feeding behavior is one area that is overdue for improvement. There is arguably no reliable and agreed upon method for measuring total food intake of flies in undisturbed, steady state conditions [2]–[5] and preference assays lack qualities appropriate for high-throughput analysis [6]. The most common methods use tracers, such as non-digestible dye, to quantify food intake and, by analysis of abdominal color, to distinguish the extent of food choice [7], [8]. Tracer methods are most effective when exposure periods are short, otherwise they report gut size rather than feeding rate [2]. Strong preference behavior is easily identified by two-dye choice assays, but intermediate preference is difficult to quantify because one must often assess different shades of the mixed color. The Capillary Feeder (CAFE) method, which requires flies to feed from calibrated capillary tubes suspended from the top of the chamber, has been proposed as a viable alternative [9]. However, it is physically challenging for the flies to access the food, which can bias data in favor of healthy flies and make long-term studies difficult. Visual assessment of feeding behavior, based on proboscis extension, has also been suggested [10], [11], but this approach is labor intensive and may confound feeding and tasting events.

Here we propose the FLIC (Fly Liquid-Food Interaction Counter), a general purpose system for accurately and continuously measuring feeding-related behaviors in *Drosophila*. The FLIC device uses a simple electronic circuit that can be monitored continuously to signal when a single fly or a group of flies interacts with a liquid food. Single flies are placed in feeding areas in which one or more sources of liquid food are provided, and they are subsequently monitored indefinitely and without disturbance. Data from each food source are collected independently, allowing for simultaneous, automated analysis of thousands of flies. We thus obtain continuous trajectories for individual flies that reflect what they eat, when they eat it, and how much they consume. For simple choices, the FLIC faithfully reproduces results obtained using traditional methods. Moreover, the system provides the power and flexibility to quantify many new aspects of feeding behavior, including temporal dynamics of food assessment and circadian feeding patterns. We envision that the FLIC system, and the principles behind it, will promote discovery in fields as diverse as aging, metabolism, and neurobiology, which require detailed analysis of food intake. It will also enable researchers to study mechanisms of feeding preference and behavior using modern, high-throughput genetic and pharmacological means.

## Methods and Materials

### Drosophila stocks

For technical reasons, short-term experiments that required starvation were done using female flies (we found that their choice patterns were more clear-cut), and longer-term experiments (e.g., 24 hr and circadian analyses) were conducted using male flies to avoid potential problems with egg-laying and to facilitate comparison with most published activity data. Unless otherwise noted, choice experiments used Canton-S female flies. Female flies carrying a loss of function mutation in the trehalose receptor *Gr5a*, *ΔGr5a*, were a gift from A. Dahanukar [12]. This mutation was backcrossed to the *w^1118^* control strain for 8 generations prior to analysis. Circadian rhythm experiments used male *Canton-S*, *yw*, and *Per^01^* flies, which carry a loss of function mutation in *Per*. *Per^01^* flies were obtained from P. Hardin [13].

### FLIC system details

**Figure 1:**
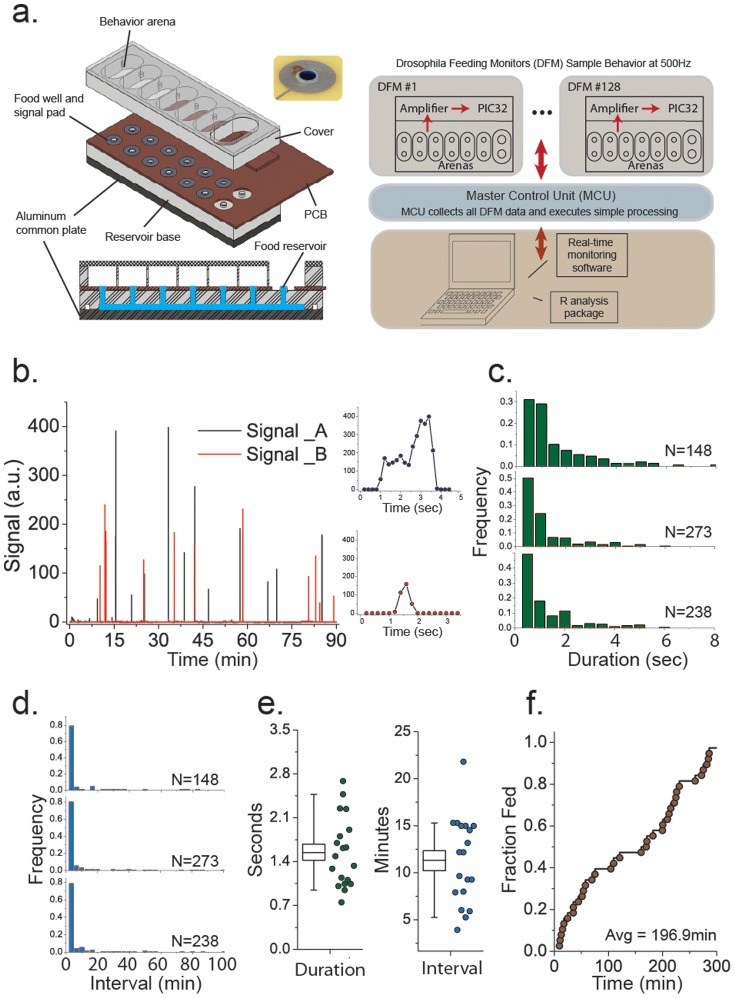
Illustration of the FLIC system. (A) Cartoon of the *Drosophila* Feeding Monitor (DFM) from the top- and side-view along with a flowchart of data collection and processing. Analog signals from all DFMs are collected by the Master Control Unit (MCU), which relays the information to the PC where the signals are visualized and recorded by the real-time monitoring software. (B) Representative signals from each of two feeding wells within a single feeding arena taken from a 90 min subset of a 24-hour feeding measurement. Close-up signal patterns representative of two distinct classes of feeding behavior events are presented as insets. (C) Histograms representing the distribution of durations for individual feeding behavior events (an event is a set of contiguous signals above baseline) over a 24 hr measurement period. Each plot represents values from a single fly, and distributions for three flies are presented. N represents the number of behavior events observed. (D) Histograms representing the size of the intervals between successive feeding behaviors over a 24 hr measurement period. Each plot represents values from a single fly, and distributions for the same three flies as in panel C are presented. (E) Among-fly variability in the average feeding duration and average time between feeding events. Each point represents the average value over a 24 hr period (N = 21). (F) Event-time distribution that represents the fraction of the population that has experienced at least one feeding at a given elapsed time from a randomized point between 12pm-2pm (N = 21). It took roughly 197 min for 50% of the population to feed at least once during this time of the day.

The FLIC system is comprised of four components (Fig. 1a). The first component, the *Drosophila* Feeding Monitor (DFM), is the physical unit that is responsible for detecting feeding behavioral events. The primary characteristics of the DFM are a set of 12 feeding wells, a dedicated signal detection circuit for each well, and a microcontroller board that controls the signal detection circuitry and that integrates data from all wells. The second component, the Master Control Unit (MCU), is responsible for coordinating up to 128 DFM, providing simple data processing, and forwarding data to the third component of the system, the PC monitoring software. The FLIC computer software allows the experimenter to control all the parameters of the system and records the data to the computer hard drive. The final component is a package for the statistical software, R, that simplifies visualization of the data and statistical analysis of feeding behavior.

The behavior arenas in the DFM are formed from an aluminum common plate, a solid plastic food reservoir base, a printed circuit board, and a plastic cover (Fig. 1a; Fig.S1). The food reservoir base is formed from 12.7 mm thick high-density polyethylene (HDPE). The twelve feeding wells, 4 mm in diameter, are placed in two parallel rows of six. Each row of wells is connected by a channel on the underside of the base, which connects them as a common food source. A large (8 mm) hole extending into each channel is provided at the end of the device to allow liquid food to be loaded and, for longer experiments, to provide an attachment point for an external food reservoir. A 6.35 mm thick aluminum plate is secured to the bottom of the reservoir base and provides a low-resistance connection between each liquid food source and, therefore, among all of the feeding wells. Elastomer O-rings around each of the two channels prevent leakage and cross-contamination of food.

A printed circuit board (PCB) is fixed to the surface of the reservoir base and serves both as mechanical support for the electronic circuitry and as a floor for the feeding arenas. Holes in the PCB achieve a press-fit around each of the individual feeding wells, which extend 0.2 mm above the floor. This design allows the liquid food in each well to achieve a modest meniscus that is easily accessible to the flies and that is isolated from the PCB floor. Surrounding each of the feeding wells on the surface of the PCB is a 13 mm diameter conductive metal pad, which is connected by standard traces to the detection circuitry.

A machined plastic cover is placed on top of the PCB to separate the floor into a number of distinct arenas depending on the design of the cover. We have constructed covers that form 6 two-choice areas (e.g., Fig. 1a) as well as covers that form 12 single-choice areas. The cover is composed of 12.7 mm thick HDPE that forms the walls of the arenas and 3.2 mm thick acrylic that is used for the ceiling. The clear acrylic allows personal observation or video monitoring of the flies during the experiments. A small hole in the acrylic ceiling above the center of each choice arena is used to insert the flies. A large opening in the ceiling at the end of the DFM allows access to the pair of food-loading holes.

To taste or consume the food in any particular well, the fly must stand on the conductive metal pad and extend a leg or proboscis into the liquid. When this occurs, the fly itself completes a simple voltage divider circuit and the resulting voltage is detected and recorded. Each individual arena houses two liquid food wells, and both foods are given a common 5 V charge through the aluminum common plate. Each metal pad is grounded through a 10 MΩ resistor, which ensures a low input voltage when the fly is not touching the food. When the fly physically interacts with the food the voltage across the 10 MΩ resistor serves as the input to a simple, non-inverting operational amplifier (op-amp) with a gain of approximately 1.2. The op-amp output voltage is detected by an 11-bit, analog-to-digital converter of a PIC32 microcontroller (PIC32MX320F128; Uno32 board from Digilent, Inc). One PIC 32 is capable of monitoring all 12 feeding wells at a rate of 500 KHz (i.e., every 2 µsec). With our circuit design, electrical current through the fly is negligible (<0.001 mA), and feeding behavior is unaffected.

The MCU coordinates data collection from each of the DFM and forwards processed signals to a personal computer for final visualization and recording. The core of the MCU is a PIC32 microcontroller, and it communicates with up to 128 DFM by Inter-Integrated Circuit Protocol (I^2^C). Each of the individual DFM employs a simple low-pass filter by maintaining a running average over a fixed number of measured signal intensities. The MCU queries each DFM several times per second to obtain and coordinates the collected data from each, and it then forwards them to a personal computer (PC) via wireless serial communication or TCP/IP. The FLIC computer software provides a real-time view of the data from each feeding well and stores signal data from each well to an appropriate text file. An open source package for the statistical software, R, provides visualization and quantitative analysis of raw and processed data. The number of active DFM in an experiment, the rate at which the DFM collects feeding signals, and the frequency at which the MCU queries each DFM are configurable.

#### Solid model and circuit design

Detailed SolidWorks part files of all machined components (metal base, plastic base, and plastic/acrylic cover) and PCB designs are provided by the authors.

### FLIC Signal Data Processing

#### Baseline calculation

Analog signals were recorded as 11-bit integers ranging from 0–1023. These values are called signal intensities, and they represent voltages ranging from 0–3.3 V. In practice, nearly all signal intensities fell into a range of 0–700. To adjust for background fluctuations in the readings, corrected intensities from each signal pad were calculated by subtracting the signal baseline. Signal baseline was determined using a median smoother of fixed window size (normally 5 minutes). Because of the high temporal resolution of the data (for the examples presented here signals were obtained every 200 ms) signal intensities that indicated feeding behaviors were rare in any given window, and the median intensities within any 5 min interval accurately represented background.

#### Behavior identification

Feeding behaviors were identified by signal intensities that surpassed a defined threshold value above baseline. For all of the experiments except those involving circadian rhythm, we used a fixed threshold. By (*i*) manually recording an exact behavior at the instance of a fly-food interaction, (*ii*) matching that to raw FLIC signals, then (*iii*) dissecting the flies gut afterward to verify a presence of blue dye to categorize characteristics of tasting and feeding signals, we established that the longer, high intensity signals corresponded to feeding events, while the ephemeral spikes were most often associated with tasting events. Thus, feeding events were defined as periods in which a particular analog signal intensity exceeded a value of 200, while signal intensities from 20–100 were considered tasting events. While we found these values to be effective for our studies, differences in the conductivity of the experimental food, for example, could necessitate an adjustment. More sophisticated algorithms are certainly possible for detecting and categorizing feeding behaviors, and we developed one for circadian behaviors, which we designed to account for modest changes in the average signal intensities that occur over the course of a multiple-day experiment. This adaptive threshold algorithm identified a significant signal as one that exceeded three times the 90% percentile of signal values over a five minute window. A minimal threshold was specified to avoid spurious events when there were zero interactions with the food in a five minute window. While the adaptive threshold performed better over the course these experiments, it does not escape our notice that there are likely more effective ways to be developed that better extract feeding information from millions of data points. Nevertheless, it is reassuring that the general characteristics of the observed phenotypes and the resulting biological inference are apparently robust to changes in the details of the analysis.

### Behavioral Assays

#### CAFE choice assays

Our CAFE assay was modified from Devineni and Heberlein [14]. The choice chamber consisted of a plastic vial with a fine metal mesh floor for air exchange and a size 0 rubber stopper capped ceiling with 2 drilled holes, which were fitted with truncated 200 µl pipet tips that allowed a snug fit for two 5 µl graduated capillary tubes (Analtech Inc., Neward, DE). The vials were placed above water to increase local humidity and reduce evaporation from the capillary tubes. Each chamber was loaded with three flies. Flies were given access to water-filled capillaries for 24 hrs prior to food choice experiments to induce modest starvation and enhance intake. Water-filled capillary tubes were then replaced with tubes filled with either 10% or 1% sucrose solution. A small amount of mineral oil was placed on top of each capillary tube to minimize evaporation. The 3 hr choice assay was performed in 25°C, 60% relative humidity, and uniform lighting. After 3 hr, the capillaries were removed and the displacement of liquid was measured to estimate the food consumption. The food displacement from each capillary tube was divided by number of flies in each vial to obtain the estimated volume consumed per fly, and preference index (PI) was calculated as “[(Volume of 10% sucrose consumed/fly)-(Volume of 1% sucrose consumed/fly)]/[Total volume consumed/fly]”. Two vials without flies were used to measure evaporation of each food solution and to adjust estimates of consumption accordingly.

#### Two-dye choice assays

We labeled 10% sucrose and 100 µM denatonium with either 0.05% FD&C #1 brilliant blue or 0.1% sulforhodamine. Each DFM was loaded with either blue 10% sucrose and red 100 µM denatonium or (the converse) red 10% sucrose and blue 100 µM denatonium. The choice assay was performed for 3 hr in the FLIC, after which flies were anesthetized by CO_2_ and inspected under a microscope to determine their abdomen color. We assigned a PI of 1 to flies with intense abdominal color matched to 10% sucrose, 0.5 to less intense color with a shade of purple, and a PI of 0 to flies with purple abdomen. Scores of −0.5 and −1 were given to flies with abdomen the color of the 100 µM denatonium food label.

#### FLIC assays

When monitoring simple feeding behaviors (i.e., for experiments that did not involve food choice), we either filled both channels of the DFMs with the same liquid food or we blocked one set of food wells with a plastic plug. In all the other cases, each of the two channels was filled with a particular food of interest. After loading the foods, an individual fly was introduced in an arena through a hole in an acrylic celling using an aspirator. We began signal collection software before flies were loaded to ensure that no signals were lost. In general, loading 8 DFMs with 48 flies took less than 5 minutes. Feeding PI values from the FLIC system were calculated as “[(Total feeding time from food A)-(Total feeding time from food B)]/Total feeding time”. See below for selection of signals generated by feeding versus tasting.

#### Behavior statistics

The duration of a feeding event was defined as the width (in seconds) of a series of sequential signal intensities above threshold. To determine wait-time distributions (e.g., the fraction fed as a function of time), a Kaplain-Meyer survival estimate was used with flies that failed to feed within a particular experiment considered right-censored. The average wait time distribution to the next feeding event was calculated, for each fly, as the time elapsed from a randomized point between 12pm-2pm until the next feeding behavior. For testing whether PI is significantly different than 0, we used paired randomization test. Measures of total consumption for each fly were computed as the sum of the durations of all significant feeding during the assay period. Linear regression analysis was used to test whether different hours of starvation can predict total feeding estimation generated by either CAFE or FLIC assay. When comparing total consumption of foods from three different starvation groups, we performed One-way ANOVA followed by Bonferroni post-hoc test.

### Circadian Analyses

#### Binning data

We used ClockLab to execute circadian analysis on the FLIC feeding behavior data. Because the resolution of our raw data is too high for ClockLab analysis, significant feeding behavior events for each fly were binned into 30 minute intervals by summing the total feeding time within that interval. Bins were defined in such a way that lights-on and lights-off occur at the junction of two bins. Binned data were output to a .txt file compatible with the ClockLab toolbox for Matlab. Transient periods of mis-communication among the DFMs, MCU, and the computer, were considered missing data, which ClockLab interprets as zero activity.

#### Normalization

Behavioral data were normalized within each fly to ensure an equal influence on the ZT plot and to avoid active individuals masking information from less active ones. Data for behavioral counts within each 30 min interval were divided by the average 30 min count for that individual over the entire experiment. A normalized behavioral count of 1 for a single 30 min interval implies an average number of interactions over that interval, 2 indicates twice the average, etc.

#### External Food Reservoir

During circadian experiments, we attached an external food reservoir to the DFMs to ensure that the liquid food was maintained at a constant level (Fig. S2a; Fig. S2b). We used a 15 mL glass scintillation vial containing the appropriate food (10% sucrose) attached to each channel by a short piece of flexible tubing. During the circadian experiments, the reservoirs were monitored under dim red light conditions and filled as necessary to maintain appropriate food levels.

#### Circadian analysis

Rhythmicity, periodicity, and power were determined using the ClockLab software as described previously [15]. Briefly, we used the power and significance values obtained using ClockLab's batch analysis functions to determine rhythmicity of individual flies. The period of all flies determined to be rhythmic was averaged to find the overall period of that genotype or treatment. Actograms present data from individual flies that were representative of the majority of flies from that genotype. They were obtained using ClockLab's scaled actogram function. Flies that died or escaped during the experiments were excluded from all analyses.

## Results

### Automated Feeding Behavior Data

The FLIC system provides an unprecedented amount of detail about a single fly's interactions with food in normal, undisturbed conditions. All of the experiments in this report used a configuration where the DFMs assess feeding signals every 2 ms. A running average over 100 signal intensities was employed to remove high-frequency noise, and the MCU forwarded processed data to the PC every 200 ms, where it was stored for future analysis. While this configuration did not utilize the full capabilities of the system, it provided sufficient resolution to distinguish tasting from feeding events (see below) without producing a crippling amount of data. Even at this limited resolution, a three hour feeding experiment using 30 flies produced 1.6 million data points. A similar-sized experiment measuring circadian feeding behavior (see below) surpassed 70 million data points.

To provide a flavor for the data produced by the FLIC system, food interactions with a 10% sucrose solution were measured for fully-fed male flies over 24 hr without disturbance or operator interference. Every significant contact between a fly and the liquid food produced a signal spike, which was visualized on the PC software and recorded (Fig. 1b). A simple threshold was used to distinguish fly-food interaction events from background noise, and interactions as brief as 50 µs were captured. We observed distinct types of events based on the characteristics of the signal including persistent signals of high-intensity as well as lesser-intensity, ephemeral spikes (Fig. 1b, inset). Low-intensity interactions were common, while sustained, high-intensity signals were substantially less frequent, resulting in an exponential distribution of duration times for individual behavioral events (Fig. 1c). For starved flies, we also observed an approximately exponential distribution of interaction times, although most events were, on average, 5 times longer than non-starved flies. We also found that flies tended to interact with the food in high-frequency bursts that were punctuated by long interludes (Fig. 1d). Among individuals, the average duration of an event in our experiment was 1.5 sec, while the average time between events was 11.3 min (Fig. 1e). Finally, the average wait time from a randomized point between 12pm-2pm until the next interaction with the food was quite long (197 min; Fig. 1f), although it should be noted that this analysis spanned the time of day when feeding behavior is thought to be least frequent (see below).

### The FLIC system vs. standard methods

**Figure 2:**
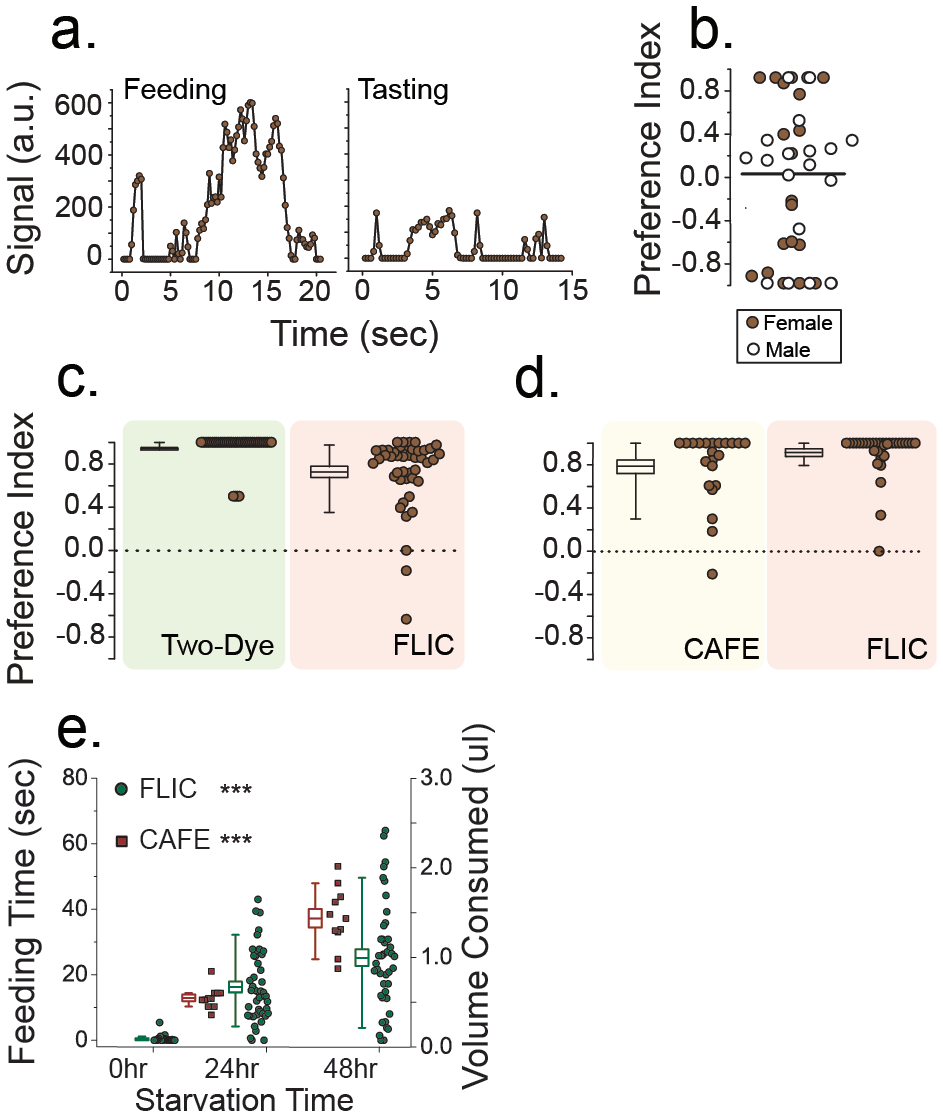
Comparison between traditional food choice assays and the FLIC system. (A) The analog signals from feeding (left) and tasting (right) behaviors have distinct characteristics. (B) When presented with identical food in both food wells, male and female flies do not exhibit a preference, which rules out systematic bias in the FLIC system (open symbol, male; closed symbol, female; pooled paired randomization test, P = 0.97). (C) Flies exhibited strong preference in favor of 10% sucrose over 100 µM denatonium when measured using both two-dye and FLIC assays (Box charts represent mean, standard error of mean, and 10–90% quantile whiskers). (D) Flies demonstrated strong preference toward 10% sucrose over 1% sucrose when measured using both the CAFE and FLIC assays (Box charts represent mean, standard error of mean, and 10–90% quantile whiskers). (E) Estimates of food consumption using the CAFE and FLIC assays. Longer starvation resulted in increased food consumption as well as total feeding time (linear regression, P<1×10^−5^ for both assays). Changes in food volume in the capillary tubes was undetectable when fully fed flies were used, and only FLIC data are presented for that treatment. *P≤0.05; **P≤0.01; ***P≤0.001.

The continuous nature of the analog signals from the FLIC system allows a broad range of feeding behaviors to be characterized. To simplify comparison with existing methods, which focus almost exclusively on total food consumption over a predefined period of time, we developed algorithms (see Methods and Materials) that categorize signal patterns into specific behaviors; longer, high intensity signals were considered to represent feeding events, while the ephemeral spikes were most often considered tasting events (Fig. 2a).

We found that inference extracted from the FLIC data using our simple algorithms was consistent with that obtained using the traditional two-dye and CAFE assays. With each of the two feeding wells in an arena filled with a different food (A vs. B), choice was quantified by a Preference Index that ranged from 1 (complete preference for food A) to −1 (preference for food B) with a value of 0 indicating no preference [14].When identical foods were placed in both wells the average PI among male and female flies was near zero, indicating that there is no significant bias inherent in the FLIC design (Fig. 2b). To establish that the FLIC system reliably identifies non-zero preference behavior we exposed female flies following 24 hr starvation to foods containing either 10% sucrose (sweet) or 100 µM denatonium (bitter). Each food was simultaneously labeled with either 0.05% FD&C blue or 0.1% sulforhodamine red (food coloring was swapped for independent experiments) to allow direct comparison with dye color measures. After three hours the flies were removed, and a PI was determined for each individual fly based on the color of their abdomen (please see Methods and Materials for detail) as well as the feeding signals detected by the FLIC system.

While both methods produced an average PI that was substantially in favor of the sucrose food, the FLIC system was able to capture greater inter-individual variability in choice behavior. Indeed, the FLIC data suggested that some flies consumed modest amounts of the bitter food (Fig. 2c), which apparently could not be detected visually based on abdomen color. To confirm that a fraction of individual flies do indeed consume 100 µM denatonium when presented as a choice against 10% sucrose, we executed similar choice experiments using starved Canton-S female flies with one modification: we added 0.5% FD&C blue dye only to the denatonium food. Following one hour during which the flies were exposed to both foods, each animal's abdomen was examined for evidence of blue, which would indicate some consumption of the denatomium-laced food. We were able to visualize blue dye in 37.5% (6/16) of the flies.

The CAFE assay is often used when both foods are palatable because different shades of mixed colors that result from the two-dye approach are difficult to quantify. We therefore compared the FLIC system and CAFE assay in their ability to assess choice between a 1% and 10% sucrose solution, both of which are known to be appetitive for starved flies [7]. For the CAFE assay, we placed female flies following 24 hrs starvation into a chamber with two calibrated capillary tubes, each filled with one of the two foods. After 3 hours, we measured the change in food volume in the tubes to calculate the amount of each food consumed per fly and the final PI. The PI estimates from CAFE and FLIC were similar in their distribution (Fig. 2d).

In addition to preference, measurement of the total amount of food a fly consumes is of interest. To illustrate how the FLIC system can be used to detect differences in overall consumption, we computed total feeding time from female flies that were starved for 24 hrs or 48 hrs as well as from flies that were fully fed. Assuming similar rates of food uptake per unit time, these estimates should be proportional to total consumption. We therefore compared the FLIC estimates with those obtained using the CAFE assay, the latter of which are based on measurements of the food volume lost in capillary tubes. To obtain a detectable change in liquid levels during a 3 hr CAFE assay, three flies were housed in the same feeding chamber, and the volume of food consumed in each chamber was divided by three to obtain per fly measures. Total feeding time (in seconds) from individual flies over the 3 hr assay was obtained using the FLIC. Despite group housing, we were unable to detect measurable changes in liquid levels for the CAFE assay using fully fed flies, while the FLIC system detected a small number of feeding events (Fig. 2e; 0 hr). Following 24 hrs and 48 hrs of starvation, a significant increase in feeding could be measured in both assays (Fig. 2e), and relative to the 48 hr values, the differences were highly consistent (an average of 66% increase in feeding every 24 hrs of starvation for the CAFE assay vs. a 50% increase for the FLIC). Notably, the distribution of data from the FLIC provides a direct estimate of the among-fly variability, and after taking into account the group measures in CAFE, the FLIC system resulted in a lower coefficient of variation (0.66 vs. 0.84, FLIC vs. CAFE, respectively).

### New dimensions of feeding behaviors

FLIC data represent feeding behaviors of variable nature and intensity as a rich set of analog signals with high temporal resolution. Having shown that simple summary statistics from these data recapitulate inference using traditional methods, we sought to propose new types of analyses that might be used to develop insight about more subtle aspects of feeding behavior. While it seems difficult to predict what kinds of hypotheses will eventually be tested using the FLIC, in this section we explore questions that interested us and that, in seeking their answers, provided a sense of the flexibility of the system and the principles involved.

**Figure 3:**
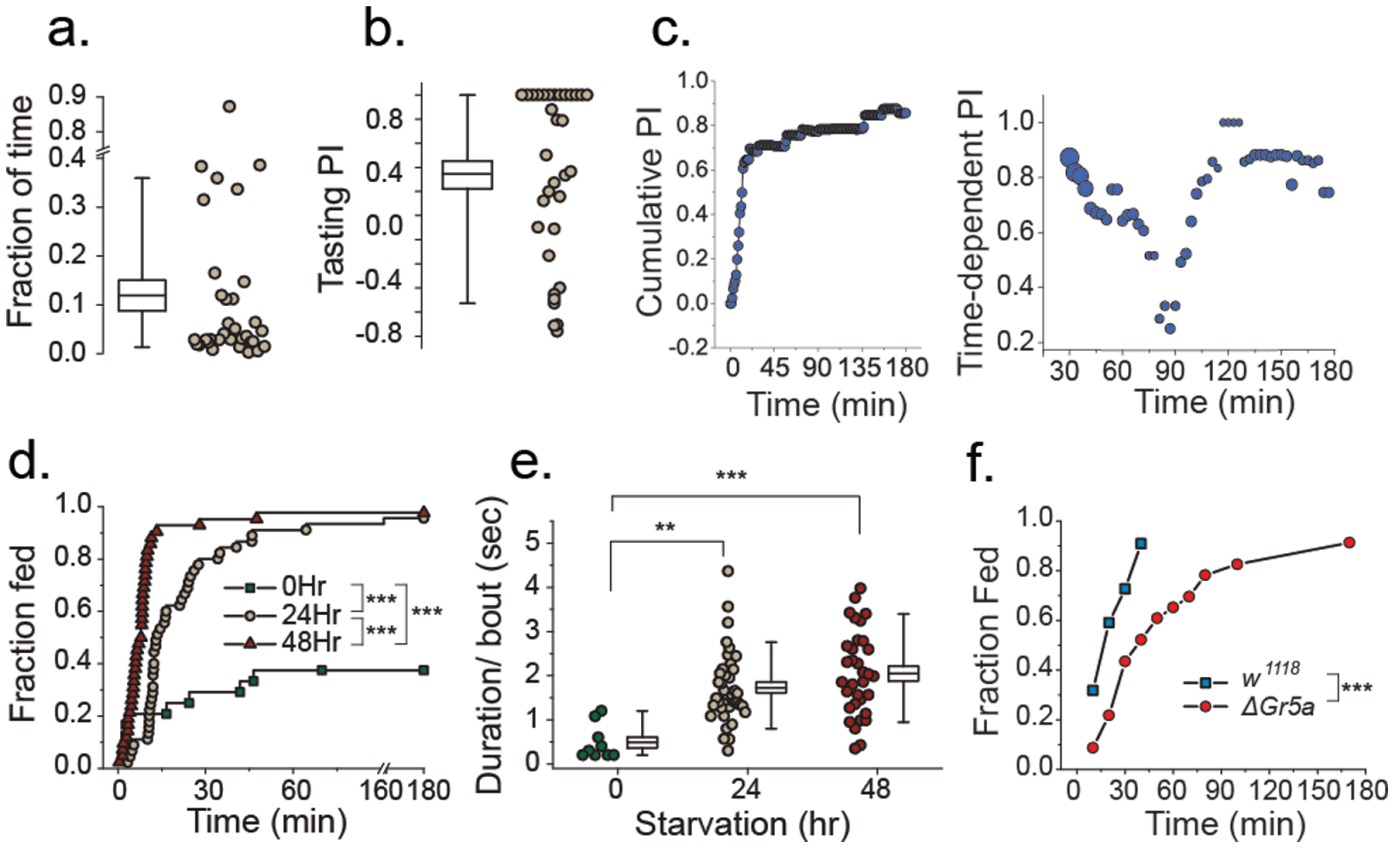
New types of behavioral inference from the FLIC system. (A) Flies spent 10% of their time in behaviors we categorized as tasting two foods prior to making their first meal choice. Fraction of time is calculated based on “total time spent tasting/time until the first meal”. (B) A greater fraction of tasting events were directed toward the food the flies choose to consume (mean Tasting PI = 0.35). A Tasting PI = 1 implies a fly tasted a single food before ultimately consuming that food. A Tasting PI = −1 implies that a fly tasted a single food before ultimately consuming the opposite food. (C) While a cumulative preference index (left panel) is effective at portraying overall feeding tendencies, time-dependent preference indices (right panel) reveal subtle differences in behavioral choices as the experiment progresses. Flies exhibited a strong preference toward 10% sucrose in the first 30 min, which was attenuated in later time periods then returned to a strong preference (N = 34; the size of the symbol is proportional to the sample size contributed to calculate PI in a given period). (D) Flies with increased feeding motivation (through longer periods of starvation) experienced their first meal earlier than control flies. Flies starved for increasing periods (0 hr, 24 hr, or 48 hr) exhibited reduced latencies until their first feeding event. Latency curves were found to be significantly different via log-rank test. (E) Flies with increased hunger (through longer periods of starvation) exhibited meals that were of significantly longer duration than control flies (One-way ANOVA followed by post-hoc test using a Bonferroni correction). (F) Taste input plays a role in motivation by decreasing latency to the first meal. Flies with loss of function in the trehalose receptor, *ΔGr5a*, were significantly delayed in their first meal of a liquid trehalose food compared with control animals (log-rank test). **P≤0.01; ***P≤0.001

How many times do flies taste each food before they discriminate between them? Can the very first feeding choice reliably predict food preference over a longer time period? Although apparently uneventful, behaviors prior to food choice may provide insight into the biology associated with sensory evaluation of the food and linked with the animal's current nutritive state [16]. To explore these issues, we calculated the fraction of time flies spent in behaviors we characterized as tasting prior to consuming their first meal in the 1% versus 10% sucrose choice experiment described above. In most cases, flies devoted less than 10% of their time to tasting prior to making their first meal choice (Fig. 3a). Remarkably, nearly 90% of the time their first meal was taken from the same food that was preferred overall during the 3 hr experiment. Flies also exhibited an increased number of estimated tasting events directed toward the food chosen for their first meal (Fig. 3b). These analyses suggest that initial ingestive behaviors result from measurable assessments and are strongly predictive of overall preference.

How does the preference behavior of a fly change during the course of an experiment? Given that feeding behavior may be driven by different mechanisms early and late in the assay [17], a method that provides a continuous estimate of preference is desirable. In such cases it is possible to calculate a time—dependent preference index, which incorporates only events that occur within a specified time window. To illustrate this principle, we used the 1% vs. 10% choice experiment to estimate a continuous preference index in which preference was calculated every 3 minutes using only the previous 30 min of feeding behavior. While the cumulative PI measure was uniformly high throughout the experiment, (Fig. 3c, left panel), the time-dependent PI measures revealed that preference for 10% sucrose changed during the experiment (Fig. 3c, right panel). Strong preference toward the higher concentration of sucrose solution was followed by a reduced preference after 90 min, which may indicate that the preference for 10% sucrose was enhanced by the importance placed on its nutritional value early on in the experiment (flies were starved prior to analysis). After satiation, the nutritional reinforcement may be lost and a lasting, but more modest, preference index is driven by taste. Notably all individuals were actively feeding when they were first introduced to the DFMs, perhaps due to hunger. After 40 min, however, often less than a half of the population were feeding over any given 30 min period, which indicates a reduction of feeding motivation after initial satiation (Fig 3c, right panel; size of symbol).

Is it possible to quantify the motivation of a fly to feed? We reasoned that highly motivated flies would feed sooner and that the duration of early feeding events would be, on average, longer. To compare feeding event data from flies with putatively different levels of motivation, we measured female flies that were starved for 24 hrs or 48 hrs as well as flies that were fully fed. We found that flies starved for 48 hours fed significantly sooner than flies starved for 24 hours and that flies from both starved groups fed significantly sooner than fully fed animals (Fig. 3d). Indeed, over 60% of the fully fed flies failed to exhibit a significant feeding event during the 3 hr experiment, while nearly all of the starved flies fed at least once during the first hour. Furthermore, the average meal duration increased significantly with increased starvation time (Fig. 3e).

Can sensory-dependent feeding behaviors be distinguished from those that are driven by hedonic or physiological reward? For example, prolonged starvation leads to preference for calorie-rich foods independent of taste inputs, while palatability determines choice under less stressful conditions [18]. Consistent with previous findings, we found that *ΔGr5a* mutant flies, who are unable to taste the sugar trehalose, demonstrated a significantly longer latency to ingest their first trehalose meal compared to control flies, consistent with the idea that a lack of taste input reduced their motivation to feed (Fig. 3f) [18]. Mutant flies did not exhibit significant differences in interactions categorized as taste behaviors prior to feeding, and they eventually developed a strong preference for trehalose, suggesting a role for hedonic feedback later in the assay.

### Circadian Feeding Behavior

The FLIC system is particularly suitable for studying areas such as circadian biology, which require long-term, continuous measures of feeding activity without disturbance. To measure circadian feeding behavior in individual flies, we equipped each FLIC monitor with an external food reservoir, which served to maintain a constant volume of liquid food in the FLIC food channels throughout the duration of multiple-day experiments (Fig. S2a; Fig.S2b). Male flies were loaded into DFMs containing a 10% sucrose solution, and the monitors were maintained under constant temperature and humidity as well as a controlled light cycle. Behavior was measured over two complete 12:12 light:dark cycles and 72 hours of complete darkness, and standard circadian analyses were applied to the data.

**Figure 4:**
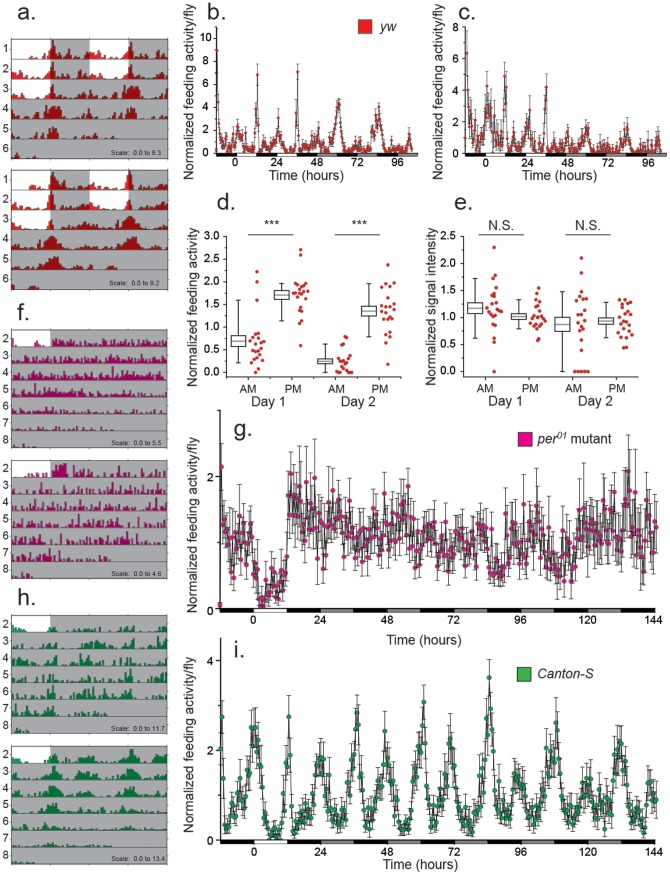
Feeding activity is circadian and dependent on the central pacemaker. (A) Representative actograms for two individual male *yw* flies depicting circadian feeding behavior during 12 h light: 12 h dark (LD) and constant darkness (DD) conditions. In all actograms, dark background indicates lights-off condition, and white background indicates lights-on conditions. Each horizontal line contains 48 hours of feeding activity data with the 2^nd^ day of data on one line repeated as the first day of data on the following line to aid visualization of circadian period. (B) Averaged normalized feeding activity as a function of time reveals a strong circadian pattern of feeding behavior. Significant behavior events were determined using an adaptive threshold (N = 22). (C) Strong circadian patterns of feeding activity persist when a conservative criterion for behavior detection is used (N = 22). (D) Total feeding activity is higher in the subjective evening compared with subjective morning. Normalized feeding activity of each fly was obtained from the 2 hour window centered on the subjective lights-on and lights-off times during the first and second days of complete darkness (two-tailed paired-sample t-test; ***P≤0.0001). (E) The frequency distributions of feeding intensity signals are not different between morning and evening times. Normalized signal intensity of feeding events for each fly were from the 2 hour window centered on the subjective lights-on and lights-off times during the first and second days of complete darkness (two-tailed paired-sample t-test; P>0.05). (F). Representative actograms of feeding patterns from two individual male *Per^01^* mutant flies. (G) Averaged normalized feeding activity as a function of time for male *Per^01^* mutant flies provides no evidence for feeding rhythms (N = 17). (H) Representative actograms of feeding patterns from two individual male *Canton-S* control flies. (I) Averaged normalized feeding activity as a function of time reveals consistent circadian rhythms for male *Canton-S* control flies (N = 22).

Circadian rhythms were clearly evident in actograms of individual *yw* flies (e.g., Fig. 4a). Indeed, 100% of the flies exhibited rhythmic feeding, with an average period of 23.3 hours and a combined power value of 64.6 ([15] and Methods and Materials). Much like general activity, feeding behaviors were concentrated near lights-on and lights-off (Fig. 4b). The periodicity remained through constant darkness, although feeding appeared to coalesce around the subjective evening at the expense of morning. This conclusion is robust to particulars of the data analysis; circadian rhythms were evident when the criteria used for detecting a feeding behavior was made significantly more conservative (i.e., a higher defined signal threshold was used), suggesting that periods of increased feeding behavior are associated with increased consumption (Fig. 4c).

Our data revealed that under 12:12 light-dark conditions and constant darkness flies feed both in the morning and the evening. While the overall number of signals indicative of feeding activity was significantly higher in a two hour window surrounding subjective lights-off compared to lights-on, the distribution of their intensities was not significantly different between the two periods (Fig. 4d; Fig. 4e). These results indicate that the types of feeding behaviors that occur in the morning and evening are similar, but that the behaviors are more frequent in the evening. To verify that flies were actually consuming significant amounts of food in the evening window, we loaded several DFMs with a 10% sucrose solution in the morning, and one hour prior to lights-off we added concentrated blue dye into the food-loading holes in the FLIC. The dye rapidly diffused throughout the food channel, thereby allowing us to introduce food tracer to each chamber without disturbing the flies. Two hours later, we found that 93.3% of the flies consumed a significant amount of dye, supporting our inference from the FLIC system.

To investigate whether the observed rhythms were circadian in nature, we measured feeding activity of *Per^01^* mutant flies, which lack a functional core clock. We found that 47% of the *Per^01^* mutant flies (N = 17) failed to exhibit any rhythmicity in feeding behavior (Fig. 4f), and the population as a whole was highly arrhythmic (Fig. 4g). When mutant flies did exhibit significant rhythms, they were weak (power  = 24.6) and widely distributed (average period  = 27.8 h, SEM = 1.86 h). It is notable that over half of the *Per^01^* mutant flies that exhibited significant rhythms had a period between 31 and 33.5 hours. However, as the rhythms are weak and are based on only 5 days of data from constant darkness, these “rhythms” are most likely the result of random fluctuation. Feeding patterns of *Canton-S* males were similar to those previously observed for *yw* males (e.g., Fig. 4b); 100% exhibited rhythmic feeding with an average period of 23.4 hours (SEM = .042 h) and a power of 67.2 (Figs. 4h and 4i) [15]. Similar to *yw* males (e.g., Fig 4d), Canton-S males also tended to feed more frequently in the evening, though the changes were more subtle and did not appear until the second day of complete darkness (Fig. S3). These data suggest that while qualitative circadian feeding behaviors are consistent across laboratory strains, genetic background must still be taken into account during these experiments.

## Discussion

The FLIC system provides a precise and continuous quantification of the number and duration of interactions a fly has with food. It complements conventional methods of analysis, such as the CAFE assay and tracer dye approaches, by allowing comprehensive long-term studies of new and subtle aspects of feeding behavior. New measures of behavior, such as the time-dependent PI, revealed temporal aspects of food choice and suggest that preference toward a particular food can be determined within any defined period. The FLIC's temporal resolution allowed an examination of the duration of each feeding and tasting bout and an exploration of the flies' level of feeding motivation. By distinguishing and quantifying both feeding and tasting behaviors in this way, it may be possible to address questions relating food palatability with the impact of metabolic or hedonic feedback. Finally, the ability to carry out long-term experiments without operator interference led to evidence that, similar to circadian changes in sensory neuron sensitivity [19], [20], feeding is prevalent both in the morning and evening and that circadian feeding is dependent on a functioning core clock. Surprisingly, we observed that a significantly greater amount of feeding in the evening, compared with the morning, which contradicts a previous report from Xu and colleagues who argued that flies concentrate nearly all of their feeding activity in the morning [21]. It seems likely that transferring flies onto the labeled food prior to data collection, as required by the protocol used by Xu et al., may have disrupted natural feeding behaviors and thereby confounded measures of food intake.

The FLIC measures represent individual behaviors and accurately capture individual variation. Although inter-individual variation in food choice is often observed by an experimenter when performing a food choice experiment, conventional methods often focus on measures of preference based on groups of individuals, mostly due to an inherent lack of resolution in the methods. For example, a preference index of 0 for a group of flies can be obtained in two ways, with either each individual in that group consuming equal amounts of two foods or by half of the flies in a group exhibiting complete preference for one food and the remaining half showing complete preference for the other. Although the latter scenario may be an extreme case, it illustrates that group measures have the potential to be unrepresentative of individual behaviors and that dominant group behaviors can effectively eliminate measurable signal from rare individuals. The FLIC system may serve as a useful tool to circumvent these issues and to better address the causes for individual behavioral tendencies.

The principles embodied in our FLIC system might be adapted to expand its scope beyond feeding behavior. For example, the DFM could be modified to deliver an electric shock upon feeding, thus providing an individual-based aversive learning paradigm [22]. Food preference could be monitored continuously and simultaneously to measure the rate and extent of learning. Additionally, it is known that flies exhibit addiction-like behavior toward alcohol [1], [14], [23]. By coupling an aversive stimulus to a fly following alcohol consumption, one may be in a position to quantify motivation for alcohol consumption in the face of punishment. In this way researchers may be in a position to observe the origin of addictive behavior and measure its strength in response to genetic manipulation.

In summary, the FLIC system is a powerful tool for dissecting context-based feeding behaviors that encompass complex interactions among the characteristics of the food and the physiological drives of the animal. By combining the capabilities of the FLIC system with genetic tools available in *Drosophila* for manipulating gene function or neuronal activity, researchers can begin to address creative questions that will reveal important insights into neuronal and molecular mechanisms regulating feeding decisions.

## Supporting Information

Figure S1 **An image of the FLIC system.** A picture of the FLIC system showing a master controller unit (MCU), four microcontrollers, and four *Drosophila* feeding monitors (DFM) that consist of six behavioral arenas and a pair of food loading holes per DFM.
(TIF)

Figure S2 **Illustration of the FLIC system with external food reservoirs.** (A) Cartoon of a DFM fitted with external food reservoirs shown from the side view (Top), the angled view (Bottom left), and the top view (Bottom right). (B) A picture of a DFM connected to two external food reservoirs.
(TIF)

Figure S3 ***Canton-S*** **males' feeding in morning and evening periods.** Beginning on the second day of DD, total feeding activity in *Canton-S* males was significantly higher in the subjective morning than in the subjective evening (N = 22). Normalized feeding activity of each fly was obtained from the 2-hour window centered on the subjective lights-on and lights-off times for each day of complete darkness (one-tailed paired-sample t-test; *P≤0.05, **P≤0.01, ***P≤0.001).
(TIF)
