# Supplementary figures and images for: FLIC: High-Throughput, Continuous Analysis of Feeding Behaviors in Drosophila
Source: PLoS One. 2014 Jun 30;9(6):e101107. doi: 10.1371/journal.pone.0101107 (PMC4076220; doi:10.1371/journal.pone.0101107)

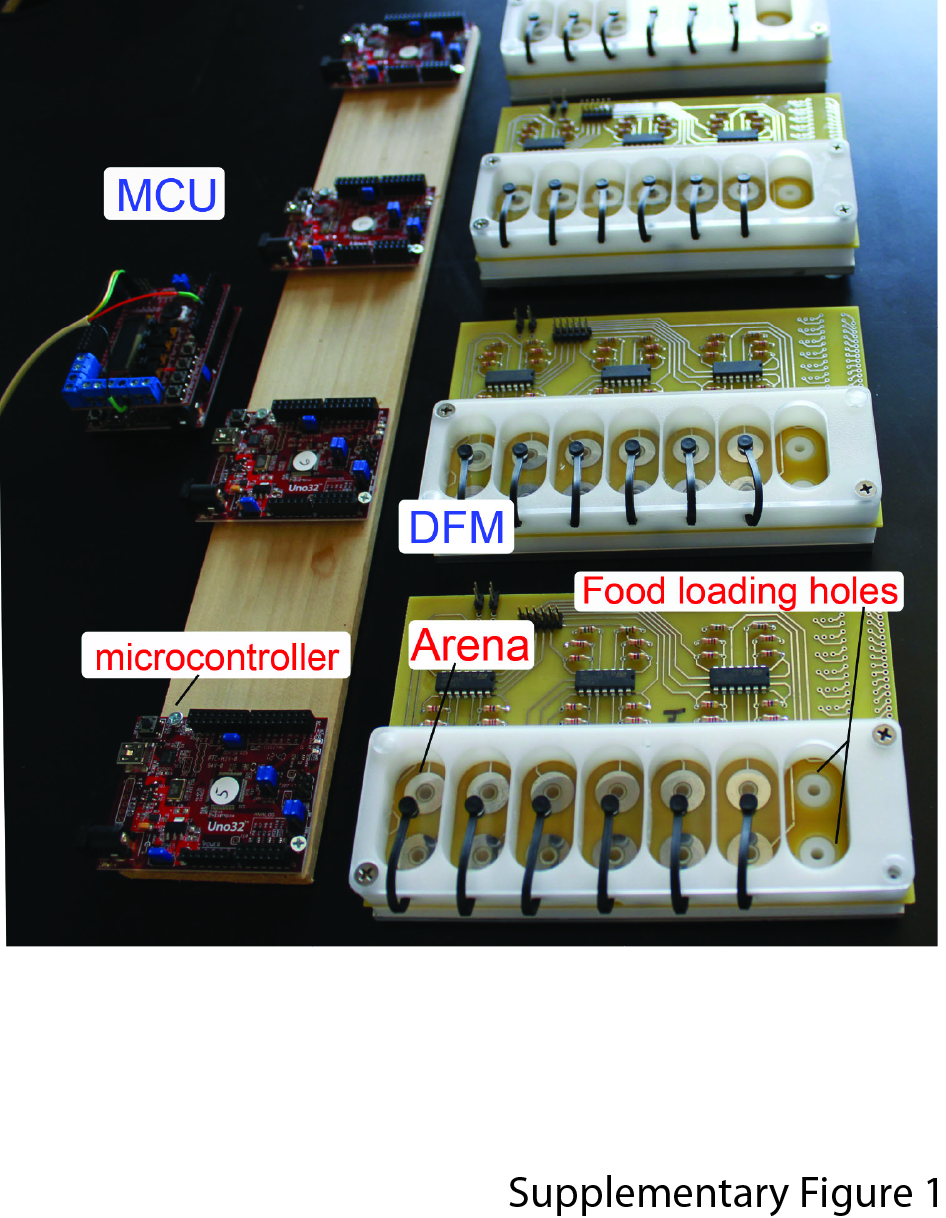

Supplement: Figure S1 — An image of the FLIC system. A picture of the FLIC system showing a master controller unit (MCU), four microcontrollers, and four Drosophila feeding monitors (DFM) that consist of six behavioral arenas and a pair of food loading holes per DFM. (TIF) [file pone.0101107.s001.tif]

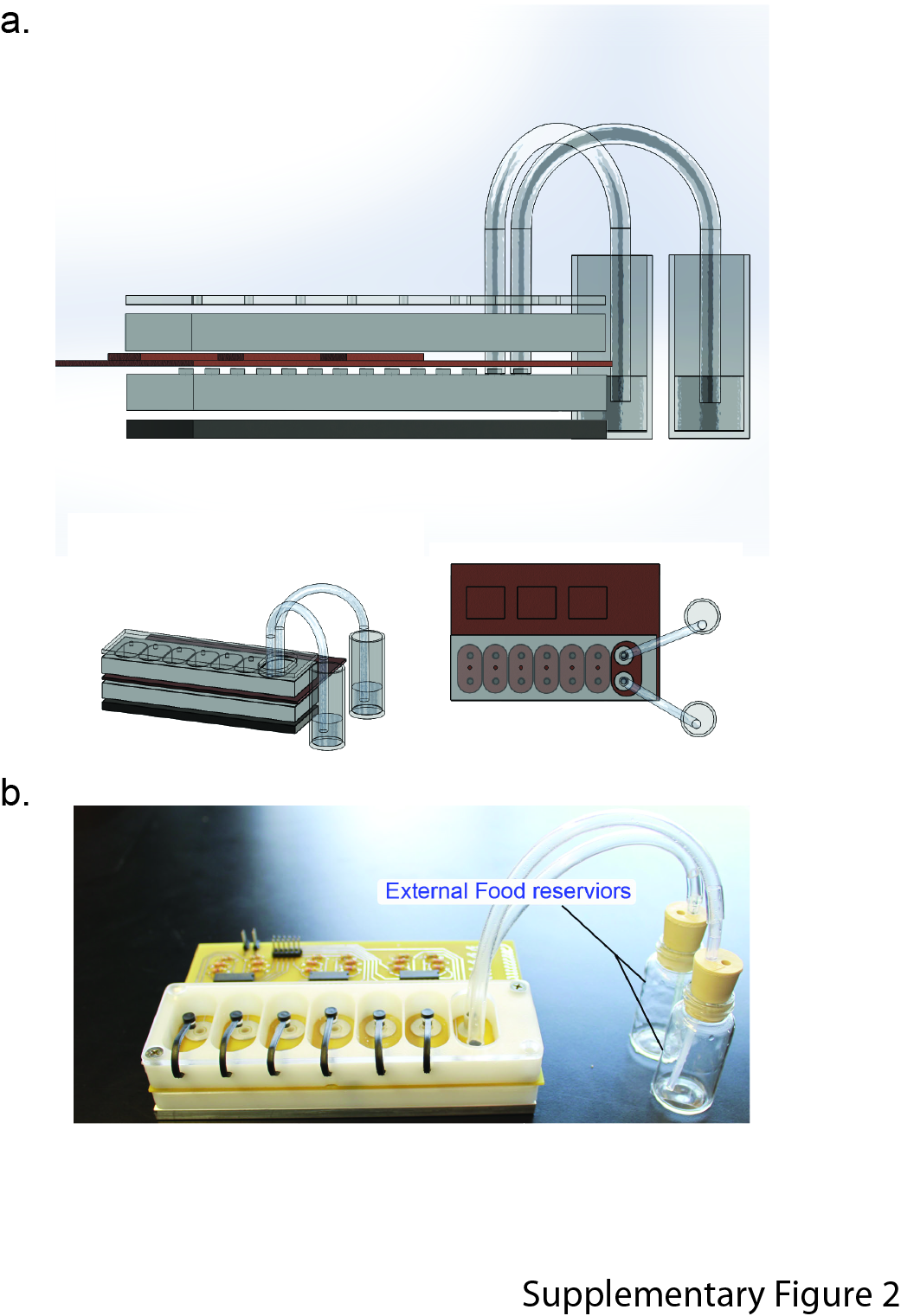

Supplement: Figure S2 — Illustration of the FLIC system with external food reservoirs. (A) Cartoon of a DFM fitted with external food reservoirs shown from the side view (Top), the angled view (Bottom left), and the top view (Bottom right). (B) A picture of a DFM connected to two external food reservoirs. (TIF) [file pone.0101107.s002.tif]

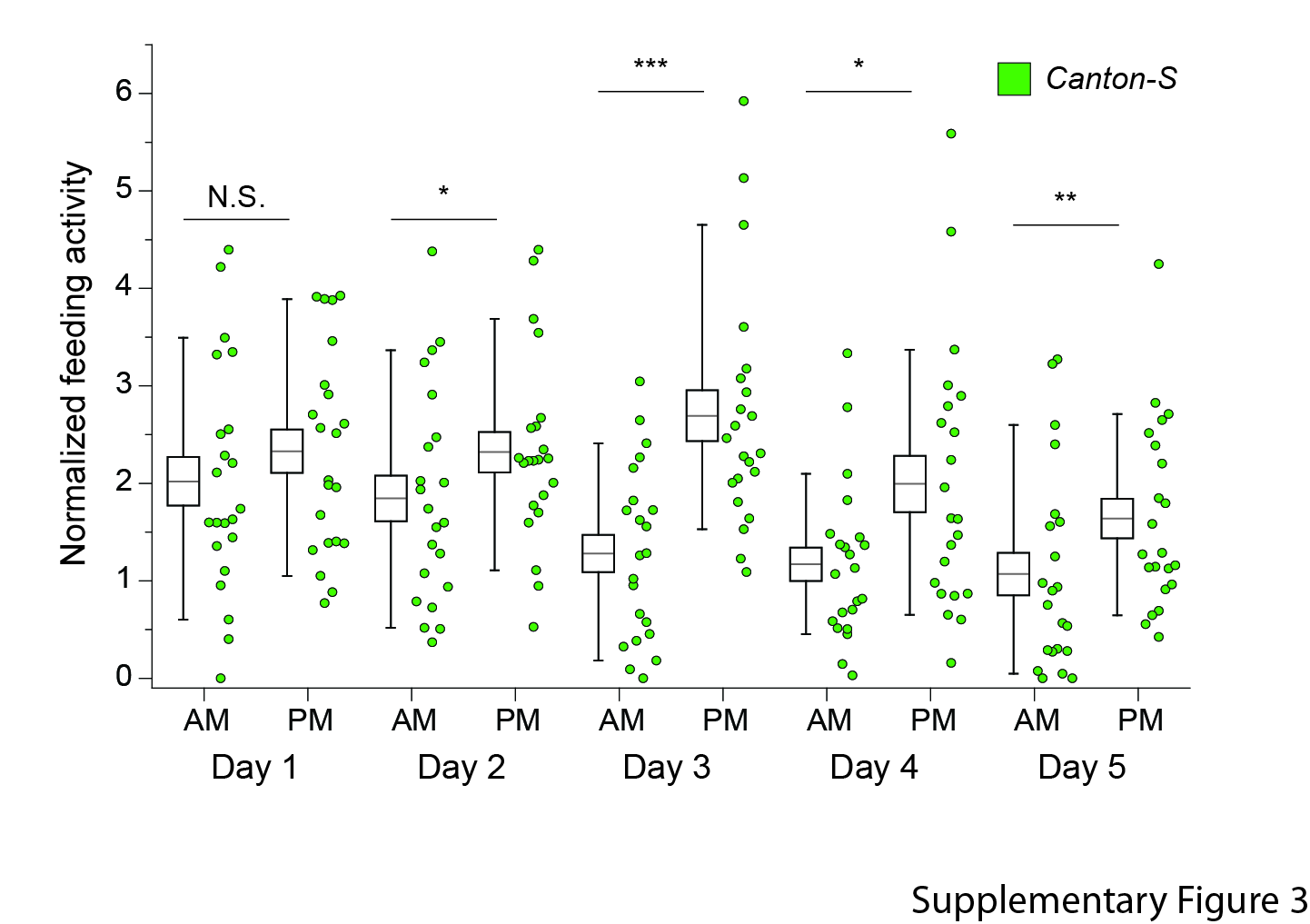

Supplement: Figure S3 — Canton-S males' feeding in morning and evening periods. Beginning on the second day of DD, total feeding activity in Canton-S males was significantly higher in the subjective morning than in the subjective evening (N = 22). Normalized feeding activity of each fly was obtained from the 2-hour window centered on the subjective lights-on and lights-off times for each day of complete darkness (one-tailed paired-sample t-test; *P≤0.05, **P≤0.01, ***P≤0.001). (TIF) [file pone.0101107.s003.tif]
